# Indigenous local observations and experiences can give useful indicators of climate change in data-deficient regions

**DOI:** 10.1007/s13412-022-00757-x

**Published:** 2022-02-22

**Authors:** Nelson Chanza, Walter Musakwa

**Affiliations:** Department of Urban and Regional Planning, University of Johannesburg, Johannesburg, South Africa

**Keywords:** Climate change impacts, Indigenous climate knowledge, Local indicators, Zimbabwe

## Abstract

This study demonstrates that Indigenous local observations and experiences can enrich knowledge of climate change, particularly in data-deficient regions that are not adequately covered by weather stations. Paradoxically, these places host groups of Indigenous Peoples who have rich knowledge about their local climates from their many years of constant interactions with the environment. The study used group-based semi-structured interviews to collaborate with keystone elderly participants who had profound knowledge and lived experiences about observed changes in their local environment (*n* = 13). These participants were identified through theoretical sampling from four remote Indigenous villages of Mbire District in Zimbabwe. The purpose of the study was to identify indicators of climate change impacts from communities believed not to have been much influenced by the scientific construct of climate change. Results revealed that the locals have a keen interest to closely observe changes occurring in their environment, including finer accounts of experiences with climatic events, owing to their predominantly climate-sensitive livelihoods. These results corroborate existing evidence about a warmer and drier climate and the reported increase in the frequency and severity of drought as well as floods in the area, and add finer details to the changes in ecological, hydrological and human systems, which are not sufficiently reported in existing climate impact studies. We also flagged new observations in biological systems as pointers for further intensive investigation. Given the complexity associated with understanding impacts of climate change and the urgent need to refine knowledge about the same, we argued for perforation of the boundaries of climate science to accommodate enriching perceptions of Indigenous communities who have been religiously observing changes happening in their local environment, albeit being relegated.

## Introduction

Existing knowledge of climate change impacts is based on coarse-grained spatial resolutions and largely generalised (Valdivia et al. 2010; Reyes-Garcia et al. 2019). Fernandez-Llamazares et al. (2017) argue that existing climate data mostly rely on records from few isolated weather stations, which are interpolated on coarse resolution grids and the methods fall short of detecting local impacts. Such gaps make it difficult to guide evidence-based climate responses that benefit local communities at risk of climate change impacts. The use of local-based knowledge, particularly in the form of Indigenous Climate Knowledge (ICK), is believed to significantly contribute in filling up this knowledge void (Balvanera et al. 2017; Reyes-Garcia et al. 2019). It is also believed that knowledge gaps are common in remote under-researched Indigenous communities, which ironically coincide with rich reservoirs of ICK that is held by Indigenous groups but largely remain untapped (Alexander et al. 2011; Cochran et al. 2013; Mistry and Berardi 2016; Garcia-del-Amo et al. 2020). The implication of this is that knowledge informing climate change regimes may remain insufficient to adequately address the unique circumstances of various communities experiencing climate change impacts. Research examining the contribution of ICK in climate change science (Speranza et al. 2010; Cochran et al. 2013; Ford et al. 2016; Reyes-Garcia et al. 2019) suggests that this problem can be addressed through bridging Indigenous and scientific knowledge. However, despite the surge in efforts to link these two knowledge systems in the field of climate science, there are mixed thoughts about the usefulness of ICK. Given the unique settings in which ICK is understood and applied, there are also many Indigenous environments whose climatic experiences remain under-reported.

By drawing on the Zimbabwean experience in Mbire District, the present study intends to comprehend the extent to which local observations of climate change and the lived experiences of Indigenous Peoples can inform climate change impacts. Stone et al. (2013) distinguish assessment of climate change from assessment of climate change impacts. The former requires significant amounts of observations over several decades in order to be able to rigorously address the detection and attribution question. Focus of this study is on climate change impacts, which are defined as indicators of changes observed by the local populations from their lived experiences with climatic processes and events. It sets the following research question: What nature, methods, and elements of climate change observations held by Indigenous populations can be used as evidence of climate change impacts? From this question, the study also determined if there were new and unreported indicators of climate change, which can guide further investigation of the occurrence of climate change. In answering these questions, the study collaborated with elderly participants chosen based on their long-time engagement in agro-based livelihoods, experiences with wildlife, fishing, and gathering of wild fruits in the area. The paper begins by reviewing current climate impact assessment studies, indicating that existing knowledge about climate change, particularly in Zimbabwe, has some shortcomings. It then describes the study area, revealing that the selected sites have a rich cultural geography, which has been fairly resilient against the evolution of rural administration and natural resources management regimes in Zimbabwe. The research methods presented embraced the multiple-evidence base paradigm that considers evidence of climate change as visible to local populations observing climatic phenomena (Reyes-Garcia et al. 2016; Smith et al. 2017). Results are discussed within this visibilist theoretical paradigm, emphasising that the contributions of Indigenous knowledge generators in climate science are equally legitimate.

## Climate change impact studies

### Multi-evidence approach to the knowledge of climate change

Despite progress in climate science, climate change impacts are not sufficiently understood. Acquisition of accurate climate data remains a challenge (Stone et al. 2013; Fernandez-Llamazares et al. 2017), particularly is data scarce regions. According to Stone et al. (2013), detection and attribution of climate change impacts is a complex process. This is because scientific knowledge on processes and mechanisms for understanding changing environmental systems has some limitations. Savo et al. (2016) express that some areas and ecosystems, for instance, will experience drastic changes that cannot be fully captured by monodisciplinary based research. Notwithstanding the improvements in the spatial resolution of global climate models, uncertainties about the local impacts brought about by a changing and variable climate are evident (Marin 2010; Savo et al. 2016). Available evidence is largely blamed for being too coarse to give accurate indicators of climate change especially in remote regions (Marin 2010; Fernandez-Llamazares et al. 2017). Marin (2010) charges that downscaled models often use scales that are not fine enough to reflect changes at lower scales, thus making predictions less effective with resource-dependent communities. Coarse-grained generalisations may not give accurate measurements of conditions and changes between recording stations. Parameters such as rainfall are likely to give errors when generalised over larger areas from quantitative measurements at recording stations (Savo et al. 2016). In addition, current assessment practices have been accused of being overly quantitative (Alexander et al. 2011; Garcia-del-Amo et al. 2020), neglecting substantial qualitative insights that have been seen by observers outside the realm of positivist scholars (Stone et al. 2013).

Against this background, there is now increasing call for the exploration of broad data sources to complement existing data in order to make climate change impact assessment more robust (Marin 2010; Alexander et al. 2011; Crona et al. 2013; Cochran et al. 2013; Ford et al. 2016; Savo et al. 2016; Fernandez-Llamazares et al. 2017). Accordingly, the use knowledge held by local people can help to give fine-grained indicators of climate change at finer resolutions (Fernandez-Llamazares et al. 2017). This is largely true in places that are poorly covered by instrumental data (Savo et al. 2016, Gurgiser et al. 2016)**.** Savo et al. (2016) opine that local accounts of climate change can give significant contributions to understanding the broad nature of climate change on ecosystems and society. They highlight that integrating local observations with global models can go a long way in explaining the social impacts of climate change instead of concentrating on physical changes alone. Advocates of this view identify various benefits of working with local and Indigenous people, which can be summarised as: critical in confirming projected trends described by global analyses of climate change (Fernandez-Llamazares et al. 2017; Reyes-Garcia et al. 2019); contributions toward filling in gaps in climate research (Crona et al. 2013; Savo et al. 2016); capability of documenting and linking direct and secondary impacts (Savo et al. 2016); better understanding of global social–ecological changes (Balvanera et al. 2017); and bringing to attention the pressing needs of local communities that are experiencing climatic impacts so as to prioritise adaptation measures (Crona et al. 2013; Balvanera et al. 2017; Reyes-Garcia et al. 2019). Some scholars raise the ethical and justice issues of neglecting contributions of Indigenous communities in climate change studies given that they have been innocent victims of climate change (Parsons et al. 2016; Smith et al. 2017).

### Climate change in Zimbabwe

Zimbabwe has a semi-arid climate system, which varies greatly across the country’s natural regions. A report by Manatsa et al. (2020) reveals significant shifts in rainfall and temperature regimes, which largely became noticeable since 1982. Since this period, the country has been experiencing changes in rainfall and temperature characteristics and related seasonal changes. Rainfall is now associated with late onset, early cessation, and contraction of the rainfall season. There is also reported increase in the number and length of dry spells and decrease in the number of rainy days. Overall, Zimbabwe has witnessed increased warming by an average of 1 °C. The drier climate is believed to cause a decline in vegetation productivity (Shekede et al. 2018; Chapungu et al. 2020; Manatsa et al. 2020). However, these impacts are not uniformly experienced but vary across the country. Existing attempts to understand these changes in Zimbabwe have only accounted for changes at regional level, that is, using the main natural regions as the bases of the analysis. For example, Mugandani et al. (2012) reveal a significant shift in natural regions, which is indicative of climate change. They indicated a shrinkage of highly productive agro-ecological regions (Region II and III) and an expansion of drier regions (Region IV and V). Manatsa et al. (2020) confirmed that about half of the country has experienced changes in agro-ecological zone categories between 1960 and 2020. Of these changes, the scholars add that about 45% experienced downgrading, while only 5% experienced some improvement. Their revised classification divides Natural Region V into V(a) and V(b) to reflect the increased aridity in this region as a consequent of climate change, where subregion V(b) is becoming drier than subregion V(a). However, the climatic changes that have occurred at local scales beyond these natural regions are still not adequately analysed.

### Gaps in Zimbabwe’s climate data

**Fig. 1:**
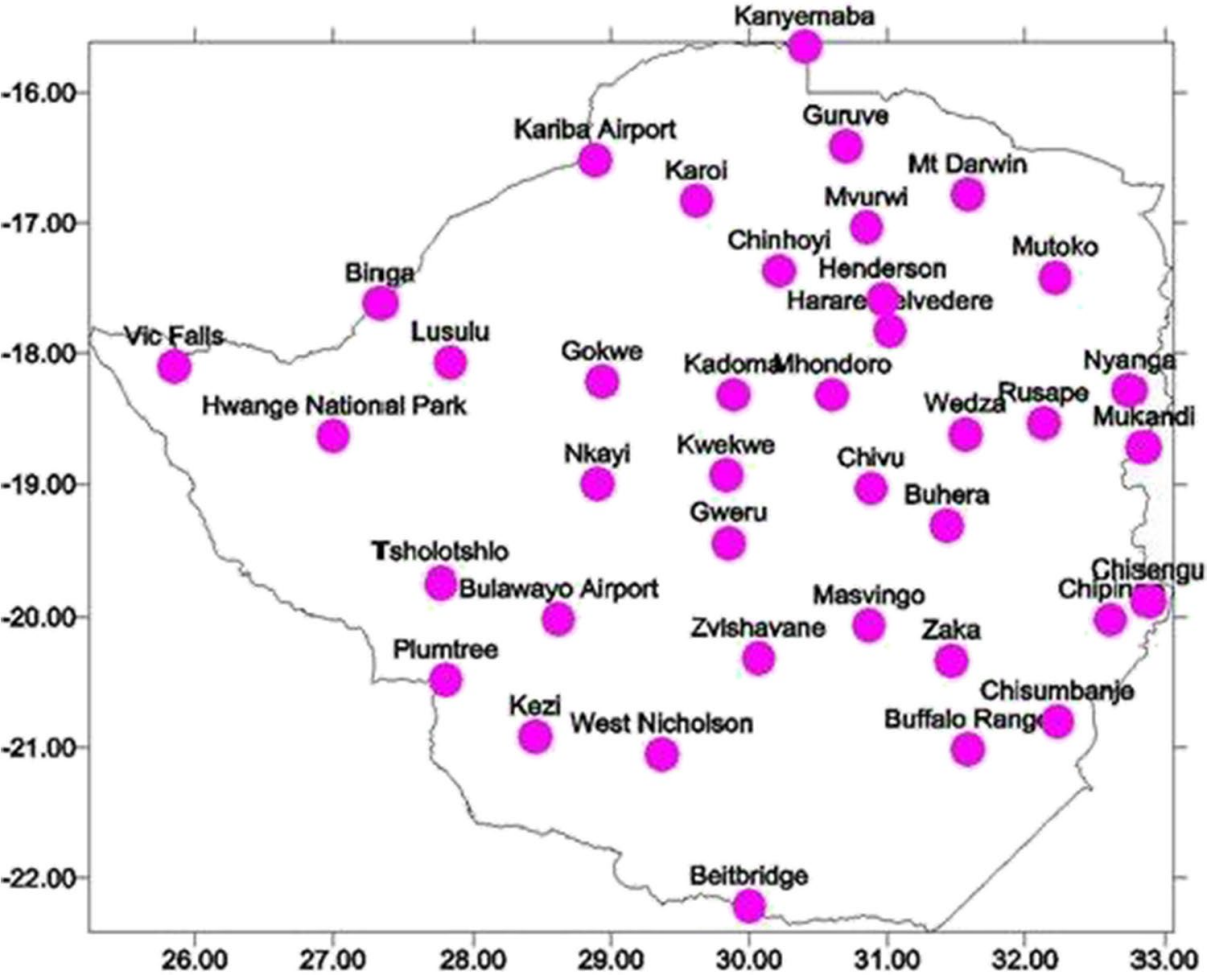
Location of meteorological stations in Zimbabwe ( Source: Mugandani et al. 2012)

Station-based meteorological data remains scant in Zimbabwe. Figure 1 shows the location of weather recording stations in the country. Given the spatial variability associated with rainfall, for example, the climate of remote places has not been evenly recorded. For example, the minimum distance between our study sites and the closest weather stations of Kanyemba, Guruve, and Karoi is about 80 km, which is above the World Meteorological Organisation’s (WMO) standards that weather stations should be within a radius of 50 km (Nhamo and Eloff 2021). These stations have been able to fairly capture data on rainfall but fall short of recording data in other parameters owing to equipment failure. Furthermore, the temporal range is hardly more than 50 years, and gaps and inconsistencies are found within the data. Quality data recording at the 45 available weather stations has been affected by obsolete equipment and limited manpower and skills in the Meteorological Services Department (MSD) associated with high staff mobility. An existing project that was supported the United Nations Development Programme (UNDP) involving installation of automatic weather stations has not improved the problem because of machine breakdown. As a result, climate data issued by the MSD is largely coarse because it is interpolated from isolated recording stations some of which use faulty equipment. The packaging of the same information and its dissemination to the farmer has also been blamed for being too technical (Rurinda et al. 2014; Gwenzi et al. 2020), making the information less relevant to the farmers. As a result, most farmers rely on their localised and tested ICK, which they perceive as more useful and reliable (Chanza and Mafongoya 2017). This suggests that ICK is utilised by communities to fill up the voids of limited climate information services. Johnson et al. (2016) allude that Indigenous Peoples in these settings have been able to maintain distinct systematic, localised, and place-based environmental knowledge for long periods of time.

## Methods

### The study area

**Fig. 2:**
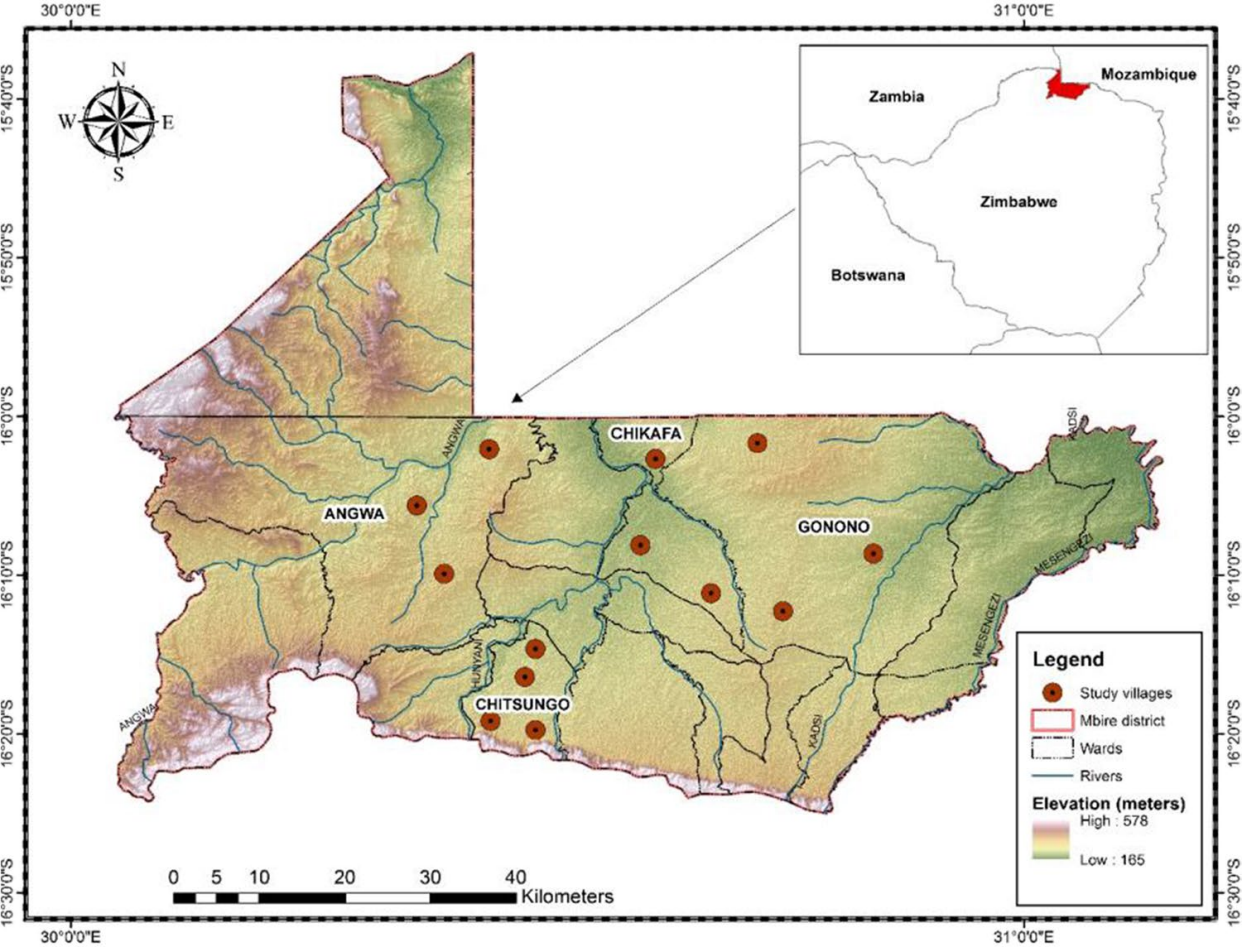
Map of the study area and study sites in Mbire, Zimbabwe

Mbire is a rural dryland district located on the northern side of Zimbabwe (Fig. 2). Choice of this study area was motivated by three main reasons: (1) It is one of the dryland regions known to be experiencing increased frequency and severity of climatic events such as drought and floods; (2) Mbire is one of the remotest districts in Zimbabwe, which is not adequately covered by meteorological stations; and (3) the area is believed to be inhabited by Indigenous populations whose long period of staying in the area could be useful to draw experiences with climatic and environmental phenomena. The major rivers that dissect the valley floor are Hunyani, Angwa, Dande, Mwanzamutanda, Musengezi and Kadzi. However, most of these rivers and other streams dry up during the dry season. These rivers flow into the Zambezi River in Mozambique, which eventually empties into the Indian Ocean. Water also collects in huge surface depressions to form seasonal inland pans during the rainfall season. These pans are a significant source of water for household use and for livestock and wildlife in the area. Mean annual rainfall is below 650 mm, while mean maximum temperature is between 28 and 30 °C (Gumindoga et al. 2020; Manatsa et al. 2020). Drought, dry spells, violent storms, seasonal flash, and riverine floods are common climatic hazards and have increased in frequency in the area.

In terms of land use, the area exhibits a montage of wildlife, settlement, and cultivation land, making it a complex socio-ecological system, which is critical in giving broad indicators of climate change under semi-arid conditions. The livelihoods of the people are quite diversified, but largely based on dryland crop and livestock farming, complemented by hunting and gathering of wild fruits. However, under the current laws, hunting or encroachment into wildlife protected areas without a permit issued by the Zimbabwe Parks and Wildlife Management Authority (ZPWMA) is criminalised. There are reports of human-wildlife conflicts emanating from crop raids and destruction of crop fields and properties by wild animals (Jani et al. 2020). Despite these problems, the locals have resiliently learnt to co-exist with the wildlife (Bola et al. 2014; Jani et al. 2020). As a way to promote the beneficial co-existence between humans and wildlife, the Communal Area Management Programme for Indigenous Resources (CAMPFIRE) was introduced by the ZPWMA in 1989.

The settlement pattern is largely linear along rivers and villages tend to be isolated. These settlements are commonly surrounded by forest stretches that are interspaced by cultivated and grazing lands. For their water requirements, the villagers rely on numerous but seasonal pools and pans. Livestock and wild animals also utilise these water sources before they dry up in the dry season. The communities have a modest leadership system involving a hierarchy of chiefs, headmen, and village heads on the one hand, while ward councillors are a parallel administration arrangement under Mbire Rural District Council (RDC) as governed by the Rural District Councils Act of 1988. The wards are the local administrative geographical boundaries. Under the traditional structures, the headman also oversees ward affairs. The wards chosen for this study are Angwa, Chikafa, Chitsungo, and Gonono. These were identified with help from the local leadership as the remotest wards where the communities are believed to be less influenced by the modern views of climate change. Reyes-Garcia et al. (2016) advise that communities believed to be less contaminated by the scientific construct of climate change are ideal for this type of study. Each of these wards has about 32 villages consisting of about 45 households. The traditional leaders have a defacto resilient customary leadership system responsible for regulating the daily activities of the community. The chiefs delegate responsibilities for the management of natural resources to headmen and village heads.

### Data collection methods

Espousing the multi-evidence approach intended to get deep insights about local indicators of climate change from the multiple sites selected, this study used group-based semi-structured elicitation interviews with elderly participants who were regarded as legitimate knowledge holders of climate change impacts. Given the remoteness of the sites used, the study assumed that the perceptions of the participants were not much diluted by the scientific interpretation of climate change. The participants selected were those who had a long history of staying in the area and were old enough to give rich accounts about their lived experiences from observing and witnessing changes happening in their environment. This was intended to give a reasonable reference period of at least 30 years that is used in climate studies. We first engaged the ward councillor in each of the selected wards who helped in identifying the villages where the participants meeting our criteria would be found. At this stage, consent to interview the Indigenous Peoples was sought from the councillors, headmen, and village heads. We used the knowledge of the local research assistants from each ward to identify the village heads who would also lead us to the desired participants. The participants were selected through chain referrals until the point of theoretical saturation (*n* = 13 groups): Angwa (3); Chikafa (3); Chitsungo (4); and Gonono (3). The groups were made up of 2 to 4 male and female interviewees, and their average age was 63 years. Overall, the total number of participants was 37 (23 females and 14 males). The joint interviews administered enabled participants to complement each other’s memories about important events that they had witnessed and the timelines of such events, and allowed peer-to-peer validation of accounts given. Accordingly, data analysis was ex ante, that is, involving participatory verification of statements given throughout the interview sessions. Each interview session lasted for about 130 min. Data were collected from 9 December 2020 to 18 January 2021.

The interview questions asked participants about the noticeable changes that they had observed in the atmospheric systems (weather and seasons, temperature, rain, wind, storms), physical system (soils, rivers, mountains), and biological system (forests, trees, animals, plants, fish), and how these changes had affected their lives and livelihoods (social system). These themes guided the subsequent thematic data analysis and presentation of research findings. Participants were also asked to state the period, direction, level, or nature of the change in the aspects mentioned and to explain what they think were the drivers of such changes. Since all the participants had spent more than 30 years of their adult lives in the area, we considered their inferences as long enough to account for observed changes in their local climate system, which is similar to the period used in climate studies. Features of interest that were referred to by the participants were recorded as detailed descriptions and/or captured in photography. In order to reflect the deeper meaning of the participants’ observations of climate change impacts, the results are largely presented as qualitative descriptions of their accounts. The interviews were administered in the local Korekore Shona dialect with the help of local research assistants from the area. The written responses were proofread to the participants at the end of each interview session to check their accuracies before they were translated into English language. Espousing the visibilist paradigm, we regarded the perceptions of climate change made by the participants as valid, consistent with emerging argument by some ICK scholars that climatic observations made by Indigenous populations are legitimately scientific (Cochran et al. 2013; Smith et al. 2017; Ebhuoma 2020). The peer-to-peer validation exercise made during interview sessions also made the findings robust. Smith et al. (2017) argued that this validation process among participants can be comparable to scientific peer review system. The study attempted to be allegiant to the views of the knowledge contributors about their perceptions of climate change indicators. However, it was also necessary to adopt reflexive pragmatism to be able to disentangle the complex viewpoints of the ICK holders into meanings that can be understood by the scientific community. Accordingly, we tried to validate some of the participants’ views by comparing with existing knowledge about climate change in Zimbabwe. Ethical clearance was granted by the University of Johannesburg under Ethical Clearance Number UJ_FEBE_FEPC_00046. There were no known ethical issues that arose from the study after observing the guidelines of debriefing, voluntary participation, informed consent, beneficence, confidentiality and anonymity, and the right to withdraw from participating.

## Results and discussion

### Observed changes in the atmospheric system

There is a wide collection of elements of the climate system that are used as indicators of climate change in Mbire. These include temperature, rainfall, mist, storms, and wind. With reference to temperature, participants revealed that the winter season (May/June/July) is becoming cooler than before. The participants recalled that long back they could not distinguish the winter season as it used to be hot throughout the year. The evidence that the winter season is now becoming very distinctive resonates with the evidence of cooler winters in some areas as reported by Manatsa et al. (2020). When asked how they were able to discern if indeed the temperatures experienced in winter had fallen, the participants confidently explained that about three decades ago they would not need warm clothing during winter season. However, there were mixed views about the general perceptions in temperature changes during other times of the year. Some indicated that they had not experienced any significant changes in temperature, while others felt that it was becoming hotter particularly during the months of September/October/November/December. Those who perceived a hotter environment indicated that they were able to tell this from observing birds dying of high temperatures, a phenomenon that they could not experience about 20 years ago. Another line of argument linked the death of birds in summer to the lack of water during this period. These accounts are consistent with the general warming of the country that was reported by Mugandani et al. (2012) and Manatsa et al. (2020).

Changes in rainfall characteristics featured prominently during group interviews. Rainfall is reported to have changed in reliability, quantity, duration, and direction of origin. The evidence emerging from the narratives of the participants is that rainfall patterns have largely been distorted. The participants described that their traditional effective rains would consistently come from the northern direction, but have become rare. Of late, the rains are increasingly becoming short-lived, patchy, and originating from any direction. The villagers also used to give distinctive names based on the time and quantity of the rains that they would receive throughout the rainfall season. They narrated that the summer seasonal calendar used to be well marked with identifiable and observable rainfall patterns that were captured in local names (Table 1). Of late, however, such rains are poorly defined and very unpredictable. The distinctive rainfall calendar would assist them to plan for the agricultural season and make appropriate farming decisions. Of late, the increasing unpredictability and unreliability of rains have forced them to abandon the practice of identifying rainfall by names.

**Table 1:** Local names used to describe rainfall types and observed changes in Mbire

Name	Description	Changes
*Runyamuti, Nhunyamuti, or Bumharutsva*	First rains received in October, which marked the beginning of the rainfall season. In the local Korekore description, *nhunyamuti* suggests that trees such as the dominant mopane would start to develop leaves after receiving these first rains. *Bumharutsva* is used to mean that the first rains would extinguish the fire scars left on vegetation burnt during the dry spell, marking the regeneration of the vegetation. These rains gave a signal to start preparing the fields for growing crops	Rains now delayed, or may not even be experienced. Sometimes, trees such as *mupane* (*Colophospermum mopane*) and *mutondo* (*Julbernardia globiflora*) begin shooting their new leaves in October before receiving the first rains
*Nhuruka*	Mostly received in November, these torrential rains marked the proper setting of the rainfall season. The villagers would utilise the moisture to start growing their crops	Now becoming highly unpredictable and inconsistent
*Zvitasiya wamera*	These rains would be received from mid-November throughout mid-December. The moisture brought about by these rains would be adequate to allow seed germination and crop growth	Becoming highly unreliable. Sometimes the farmers plant maize and sorghum seeds but the seeds may fail to germinate owing to insufficient moisture
*Mubvumbi*	*Mubvumbi* refers to drizzle that was mostly experienced late December throughout early February. The rains would take up to 7 days. The duration and spread of these rains during this period would be linked to the maturity of cereals (maize and sorghum). If the *mubvumbi* is prolonged to February, they knew that crop yields would be good	It is now rare to experience continuous rains lasting up to 3 days. The months of January/February are becoming drier
*Chihore*	These were very light rains received in March/April, but effective to allow the maturity of late season crops	It is now rare to get rains in March/April

The rainfall season is also understood to have significantly shrunk as measured by the late start of the rains and the early termination of the rainfall season. The traditional rainfall onset dates are reported to have shifted from mid-October to December, with early cessation of the rains about a month earlier in February/March, instead of April. Apart from the rainfall season becoming shorter, it is also noticeably and commonly becoming drier as a result of shorter rainy seasons. Drought is becoming a common feature every agricultural season, and the season is reportedly being punctuated by increased number and length of dry spells. They now experience more drier days in the months of January/February. The participants were able to associate this increased dryness with the wilting of their crops around this period. Effective rains that would signal the onset of the proper farming season were said to be a rarity. These rains would be experienced around mid-October or early November, if the rains are delayed. According to the interviewees, if these rains are received, they are now coming lately around mid-December. The participants added that if significant rain downpours are received in December/January, the rain would usually be violent. Such short-lived violent storms, which the people named *chimvura mupengo* or *gumbura*, if they lead to flash floods, are usually accompanied by heavy winds. These terms suggest that the storms are very destructive. Evidence suggests that over the past 10 years there has been an increase in violent tropical storms and cyclones in the area (Mudavanhu et al. 2020). The participants explained that they “used to get into the Christmas Holiday when the maize crops would be at tasselling and silking stage, but of late (they) can get into the New Year while the fields are bare.” For participants in Chitsungo, the mist that used to cover Mushongavende Mountain, a stretch of the Zambezi escarpment, is now rarely witnessed. The mist used to be an indicator of imminent rains. The participants recalled that the changes in rainfall became evident since 2002. These statements about the changes in rainfall support the observation that the climate in Zimbabwe is becoming drier because of little rainfall (Serdeczny et al. 2017; Manatsa et al. 2020). The detailed accounts in the rainfall regime that were provided by the locals enrich our understanding about rainfall characteristics as similarly reported by Marin (2010).

### Observed changes in the physical system

**Fig. 3:**
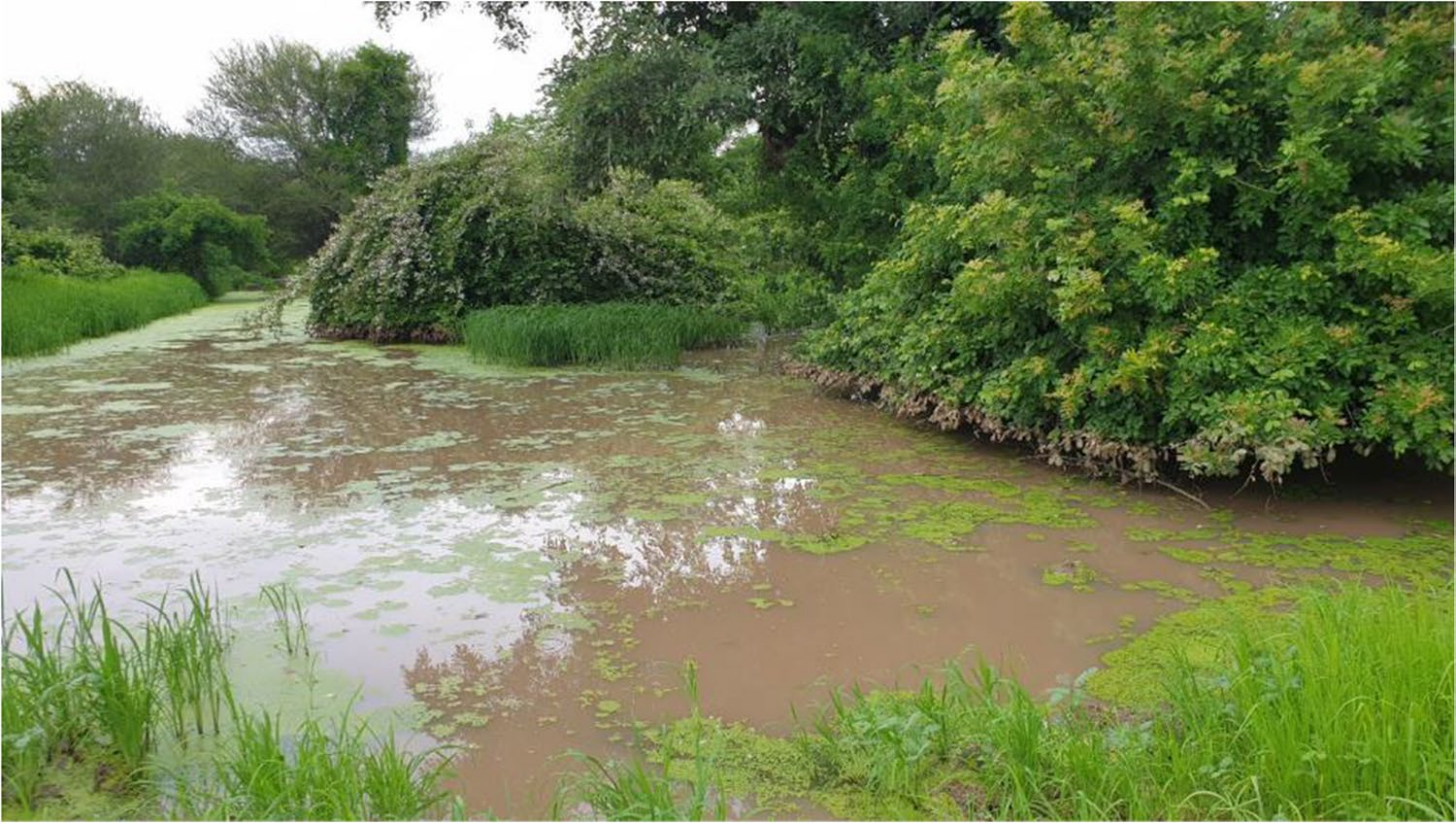
Nyagwena pan in Chitsungo, which used to keep water throughout the year but now dries up in June. (Image taken on 3 January 2021)

There was a common observation by the participants that most of the rivers that used to be perennial have become either seasonal or ephemeral. For example, Hunyani and Angwa rivers that used to flow all year round are now seasonal, while Irira River has become ephemeral. Hunyani River whose channel used to be narrow and deep has widened into a shallow sand filled channel that dry up as early as August. The participants attributed the increased frequency and severity of riverine flooding to the combined effects of river channel disturbance and heavy storms. Deep pools like the sacred Mushongavende found in Hunyani River have become smaller and shallower. Other pools such as Nyamoto and Nyamvuu that used to keep hippopotamuses and crocodiles are now drying up in August/September before the onset of the next rainfall season. However, climate change could be reinforcing other environmental stressors associated with upstream farming activities. The interviewees also revealed that there has been increased desiccation of *makambwe* (pans) as evidenced by the reduced time they keep water. Some of these pans that used to keep water throughout the year are said to be drying up early in May/June (Fig. 3). The participants believed that the combined effects of low rainfall and livestock and wildlife populations are behind the drying up of the pans. Gumindoga et al. (2020) attributed the desiccation of water sources to the high rates of evapotranspiration in the area.

Participants unanimously attributed the increased degradation of river courses to the activities by both humans and animals as they adapt to a drier climate. In one of the interviews conducted in Chitsungo, the interviewees stated:

The increased concentration and roaming of cattle along Hunyani River banks for both water and reeds during dry season has induced severe degradation of the river channel. We are now establishing our gardens along the river banks to access water from the shallow wells that we dig in the bed of Hunyani River. This will be the only source of water for animals and people during the months of September/October/November. However, elephants also come to the river to raid our gardens. (Group interview with 3 participants, Chitsungo)

The villagers in Gonono who mostly obtain their water from wells revealed that the water table has gone down owing to drier conditions being experienced. “The water that used to be accessed at depths of 8 m around the late 1990s can now only be accessed if one digs further down to depths of 26 to 30 m. People and livestock are now sharing ground water from these wells” (group interview with 2 participants, Gonono). Kusangaya et al. (2013) revealed gaps in understanding the hydrological responses to climate change in Zimbabwe. The site-specific details about impact of climate change on water resources that these results show can be used to guide adaptation decisions.

### Observed changes in the biosphere

A number of indicators of climate change are quite evident in changes in abundance and behaviour of wild animals and birds, changes in the phenology of plant species, changes in fish species, and the decline in bees. The drying up of water bodies seems to be coinciding with the disappearance of hippopotamuses and decline in crocodile numbers. However, the latter were reported to be increasingly attacking livestock. The participants also indicated increased incidences of crop raids by elephants, baboons, monkeys, and wild pigs, thereby exacerbating human wildlife conflict. In Angwa, for example, participants complained that, “Elephants were now eating tomatoes and onions from vegetable gardens, a strange event in the area.” Furthermore, stories about the increased attack on goats and chickens by hyenas and baboons were repeated in all the villages studied. To the participants, it was not clearly evident if indeed these animals had increased in numbers, but most of them argued that what they could certainly tell was the change in behaviour of the animals. They believed that the increased crop raids could be attributed to drought-induced food shortages, which is largely associated with habitat shrinkage as a result of both climate change and human activities. For example, it emerged in one of the interviews held in Gonono that, “It has become common to see animals such as zebras and kudus coming to the villages during daylight desperately seeking water.” It was also indicated that livestock attacks by crocodiles in Hunyani River tended to be higher during the dry season, which they associated with the concentration of cattle and goats to the only water source remaining during the dry period of the year. What makes the situation worse in Zimbabwe is that there are no laws that support compensation to local communities as a result of wildlife attacks. Jani et al. (2020) link the rise in cases of human-wildlife conflict to the increase in elephant populations as a result of conservation efforts. Drought could also be worsening the conflicts as the animals uncontrollably roam around in search of dwindling food sources.

The changes associated with pests and disease incidences have not been adequately documented in the country’s climate impact research. Participants’ narrations pointed towards a surge in incidences of diseases that affect humans, crops, and livestock, including increases in new insects and pests. Although they mentioned an increase in malaria as a result of warmer temperatures and other diarrheal diseases to be affecting the people more, the interviewees stated that there are some unknown ailments that were not experienced before. The participants related the surge in these diseases to water shortages. They also associated the pests and diseases with drought and the general change in ecological conditions. Armoured crickets (*mamunye*), fall army worms, and millipedes were reported to be becoming increasingly notorious as crop pests, while there has been a reduction in mopane worms (*Gonimbrasia belina*) and grasshoppers. The interviewees indicated that baboons were consuming these worms, a phenomenon they regarded as unusual. The decline in grasshoppers could be caused by the drought-induced reduction in vegetation as reported by Chapungu et al. (2020), or other form of predation that the participants had not closely noticed. Although not much was reported about changes in aquatic species, some interviewees were of the view that certain fish species (tilapia, *mukupe, muchenyu)* that used to be plenty in rivers were now rarely found. Only those species (catfish, eels, *karangezhi*) that appeared to adapt well to the new conditions were becoming more common.

Migratory stock birds such as the once popular *shuramurove*, whose appearance was interpreted by the villagers to mark the onset of the rainy season, are now rarely found. The interviewees indicated that such birds used to be found every year in October/November. They also reported about common incidences of birds coming to courtyards to look for water particularly during the dry season when most ground water sources would have dried up. Honey harvesters used to come across the honey bird (*tsoro*) whose sounds and movement were decoded by locals to track the location of bee hives. However, the interviewees indicated that it was becoming rare to come across such birds and the bee hives. This observation resonates with conclusion by Giannini et al. (2020) that climate change negatively affects bee populations, posing serious consequences on food security.

Participants also revealed that they keenly observed changes in plants, particularly fruit trees that they rely on as supplementary food sources. Wild fruits that used to be plenty, such as *manyanya (Dioscorea steriscus*), *tsvanzva* (*Ximenia caffra*), and *mhande* (a cereal-like grass harvested and pounded to make mealie meal), were reported to be declining. The fruiting of wild fruit trees was reported to be becoming irregular, with fruits such as *masau (Ziziphus mauritiana*)*, matamba (Strychnos spinose*)*, mauyu (Adansonia digitata*)*, hacha (Parinari curatellifolia*)*, chenje* (*Dyspros mespiliformis*), and *siga (Tamarindus indicus*) said to be declining in both size and abundance. They have also observed a reduction in the size of Acacia tree pods, which are eaten by livestock during the dry season when food becomes scarce. The participants also revealed delays in the flowering of such trees as *Ziziphus mauritiana* and *Tamarindus indicus.* They attributed these irregularities to drought. Encroachment of some areas by invasive alien species was also indicated. For example, a rhizomatous weed that the locals referred to as *ndawe,* including *bongonwe* grass now commonly found in floodplains are believed to have been dispersed by floods. In some places such as Gonono, thatching grass is said to be becoming shorter owing to drought and overgrazing by animals. Although it is difficult to directly attribute these changes to climate change, a study by Reid (2016) revealed that the precision of narrations given by Indigenous populations could support ecological research and monitoring in climate change studies.

### Changes in the social system

There were reported adjustments in the villagers’ livelihoods and way of life, which are indicative of the need to suit the new changes in their local environment. The locals have changed their farming practices largely because of drought and animal raids on crops. The dual season cropping that they used to practise is no longer supported by a drier climate. Riverine residual moisture that used to support off season farming activities, where new crops would be planted in April/May after harvesting the first summer crop, is no longer feasible. For example, participants in Chikafa explained that, “Winter maize cropping has since been abandoned because moisture is no longer enough to take the crop to maturity.” They added that livestock spent much of the time along the Hunyani River, where there would be water and green reeds, which makes it difficult to grow the winter crops. Although Gumindoga et al. (2020) indicated suitability for flood recession farming in most of the areas closer to rivers, as a way of adapting to moisture shortages in Mbire District, the experiences by the local farmers reveal adaptation constraints emanating from crop damages by elephants.

Consistent with other studies by Dube et al. (2017) and Mubaya et al. (2017), this research also recorded changes in division of labour and gender roles. The frequency of watering gardens was reported to have increased during the dry season. People now have to travel longer distances to access water. Given the long distances travelled to get to the gardens and get water for domestic use, there are instances where the villagers worry about girls’ safety. This highlights the gender impacts of climate change where women often felt a much heavier burden than man (Tanyanyiwa and Mufunda 2019). In such cases, the girls would either be accompanied by boys or go to the river in groups. On the other hand, young men and boys either have to accompany livestock to get drinking water from the pools, or they would need to rely on water from the wells that they give the livestock during the dry season. There is also evidence of deserting land perceived to be dry in favour of riverine farming. The shifting labour requirements (Dube et al. 2017; Mubaya et al. 2017) also shown by this study are an emerging issue requiring closer analysis.

## Conclusion

The evidence of climate change reported in this study has overly been qualitative, lacking in the quantitative assessment of the climatic elements and events. However, the results of this qualitative analysis were consistent with available evidence of climate change reported in Zimbabwe. These findings have also confirmed the existence of climate change experienced by locals in places which have not been evenly covered by meteorological data. The peer-to-peer validation process also made the findings robust enough to give credible information about climate change in the study area. An important observation emerging from this study is that Indigenous local observations and experiences can give useful indicators about the existence of climate change phenomena. Such indicators are quite informative especially in places with climate data deficiencies. The finer accounts from the narrations of ICK holders are also capable of enriching the knowledge of climate change impacts. The richness of this evidence is made possible because or two main reasons: (1) the Indigenous knowledge generators have had lengthy periods of closely observing changes happening in their local environments, and (2) because their natural resources based livelihoods are directly dependent on climate, they have a keen interest in observing climatic phenomena and their lived experiences with climatic observations give them profound memories about past events, including how they have evolved under such conditions.

The indicators identified in this study cover the broad areas commonly reported in climate impact studies, which include atmospheric, hydrological, biological, and social elements. Overall, our study confirmed existing reports about a warmer and drier climate system associated with general increase in temperatures and precipitation deficits. Thus, our study highlights the utility of ICK in the absence of regularly collected meteorological data. Our study is also spatially explicit as it does not generalise but highlights changes in a particular area. Added to this are the reciprocal changes in physical systems such as irregular water flow regimes and silting of water bodies, and the subsequent decline in aquatic species. When witnessing these changes, the local people do not just passively observe climate change impacts but devise location-specific responsive measures that enable them to continue to exist under the harsh environments. Identifying these local indicators is crucial in ecosystem-based adaptation that can be applied elsewhere. Through experimentation and trials, the people learn about adaptive mechanisms that are useful in understanding the social and cultural dimension of climate change impacts. Within the social dimension, there are also cultural practices (e.g., the practice of assigning names to rains) that people are abandoning, which could lead to the erosion of Indigenous cultures. The precision in describing the nature, magnitude and directions of the changes, including the site-specific descriptions of the impacts being experienced can be used to guide adaptation choices and planning. However, there are also attribution challenges that require careful probing and reflexivity to be able to separate climatic impacts from anthropogenic drivers of environmental changes, although the latter could be related to indirect inducers as people try to adapt to the impacts of climate change.

Essentially, the information given from this study does not only enrich climate change knowledge, but has also unearthed new evidence pointers, which can potentially be used to guide further climate impact assessment studies. This is an opportunity for the integration of ICK with scientific knowledge to enhance understanding of climate change. There are many signs in the ecological system particularly, the emerging dynamics and complexity of human wildlife conflicts associated with the increasing pestiferous nature of wildlife owing to the dwindling food sources for both humans and wildlife, which could be climatic. It is therefore plausible to conclude that the boundaries of climate change impact assessment should be opened up to accommodate the participation of ICK generators as a way to enhance understanding of climate change science. In line with new paradigm requiring that Indigenous Peoples receive benefits from the research in which they participate, this study considers getting back to the villages to share the findings once the COVID-19 situation would permit.

## Data Availability

The data that support the findings of this study may be available on request from the corresponding author, NC. These data are not publicly available because of agreements made with the participants, who are the custodians of their Indigenous knowledge as determined by both research agreements with the indigenous communities, as well as university ethics approval.
